# Supplementary material for: Machine Learning for Predicting the Low Risk of Postoperative Pancreatic Fistula After Pancreaticoduodenectomy: Toward a Dynamic and Personalized Postoperative Management Strategy
Source: Cancers (Basel). 2025 May 31;17(11):1846. doi: 10.3390/cancers17111846 (PMC12153646; doi:10.3390/cancers17111846)
Supplement: Supplementary file 1 [file cancers-17-01846-s001.zip › cancers-3651399-supplementary.pdf]

Supplementary Materials

Feature Correlation Analysis

To explore potential multicollinearity and variable interactions among the features used in the model, we computed the Pearson correlation matrix for all available predictors. The heatmap presented in Figure S3 highlights the pairwise correlations between clinical, microbiological, biochemical, and procedural variables.

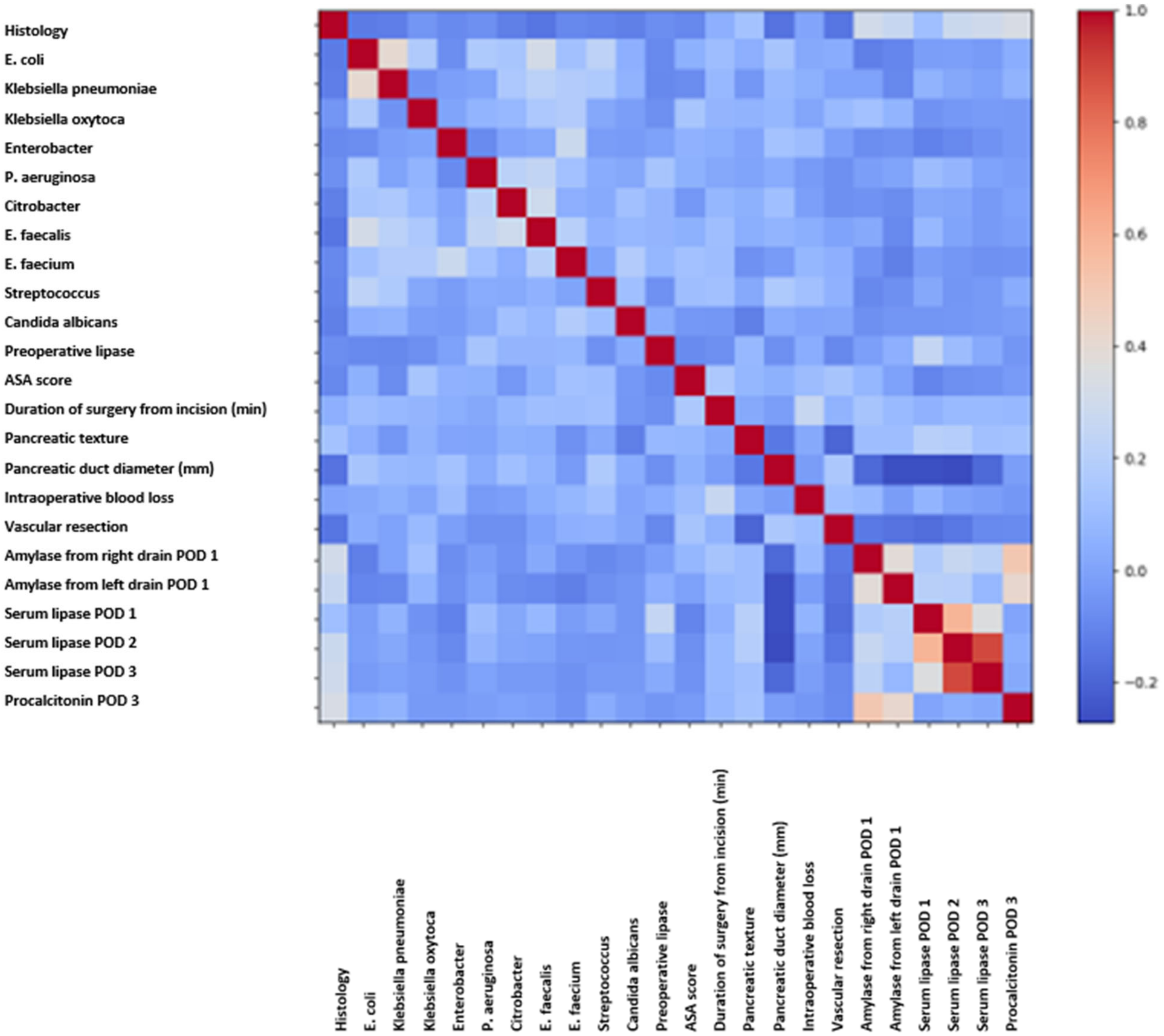

Figure S1. The heatmap visualizes pairwise Pearson correlation coefficients among all features used in the predictive model, including clinical, intraoperative, microbiological, and postoperative biochemical variables. Warmer colors indicate stronger positive correlations, while cooler shades reflect weak or negative associations. Strong correlations are primarily confined to early postoperative inflammatory markers (e.g., PCT and lipase levels across PODs), while other features demonstrate low interdependence, indicating good diversity in the predictor space.

Calibration and Decision Curve Analysis

To evaluate the reliability and clinical utility of the model's predicted risk estimates for drain removal decisions, we performed calibration analysis and decision-curve analysis (DCA) in a multiclass setting, considering the three clinically relevant outcomes: No Fistula, Grade A Fistula, and Grade B–C Fistula.

Calibration Curves

Supplementary Figure S2 shows the calibration curves for each class using a one-vs-rest strategy:

- **The No Fistula class (left panel) demonstrates excellent calibration, with predicted probabilities closely matching the observed frequencies across the range of predictions (Brier score = 0.045).**
- **The Grade A (middle panel) and Grade B–C (right panel) classes exhibit more variability, particularly at intermediate and high predicted probabilities. This may be attributed to class imbalance or limited sample support in certain bins, though the overall Brier scores (0.158 and 0.140, respectively) remain acceptable for probabilistic interpretation.**

These results indicate that the model's predicted probabilities are generally well-calibrated, particularly for the most prevalent class (No Fistula), and provide a reasonable approximation of the actual risk.

Decision-Curve Analysis

Supplementary Figure S3 presents the decision-curve analysis (DCA) for each outcome. The net benefit was calculated across a range of threshold probabilities to assess the clinical value of using the model for decision-making:

- **The No Fistula model provides the highest net benefit across most thresholds, confirming its utility in identifying low-risk patients who could be candidates for early drain removal.**
- **The Grade A and Grade B–C models also yield positive net benefits at lower thresholds, suggesting some value in identifying higher-risk patients, though the benefit diminishes at higher thresholds, likely reflecting greater uncertainty in predicting rarer outcomes.**

Overall, DCA supports the integration of the model into clinical workflows, particularly to safely reduce unnecessary drainage in low-risk patients while still capturing clinically significant complications at the higher end of the risk spectrum.

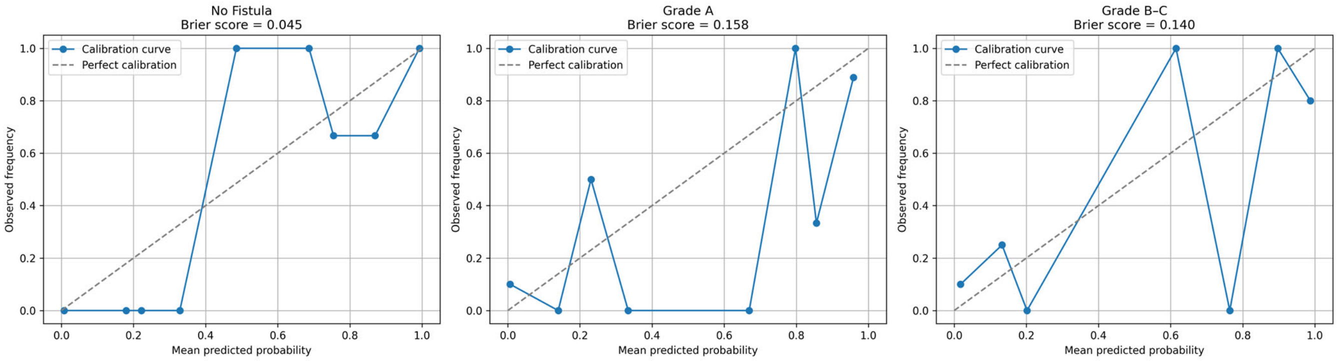

Figure S2. Calibration plots are shown for each class using a one-vs-rest strategy: No Fistula (left), Grade A (middle), and Grade B–C (right). The x-axis represents the mean predicted probability for each bin, and the y-axis shows the corresponding observed frequency. The dashed diagonal line indicates perfect calibration. The Brier score, reported for each class, summarizes the accuracy of probabilistic predictions. The model demonstrates good calibration for the No Fistula class, while predictions for Grade A and Grade B–C exhibit greater variability, likely due to lower class prevalence.

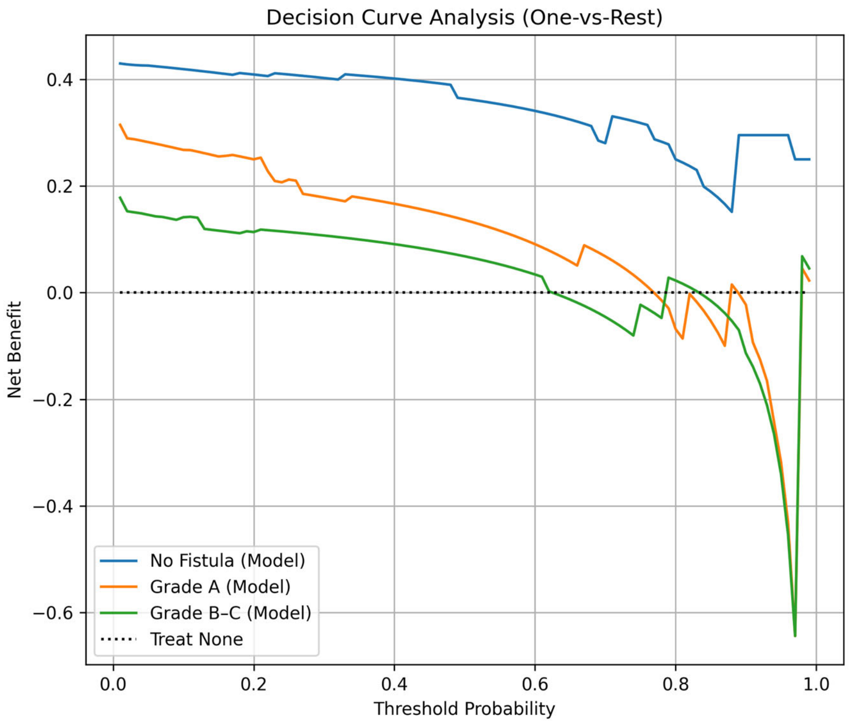

Figure S3. Calibration plots are shown for each class using a one-vs-rest strategy: No Fistula (left), Grade A (middle), and Grade B–C (right). The x-axis represents the mean predicted probability for each bin, and the y-axis shows the corresponding observed frequency. The dashed diagonal line indicates perfect calibration. The Brier score, reported for each class, summarizes the accuracy of probabilistic predictions. The model demonstrates good calibration for the No Fistula class, while predictions for Grade A and Grade B–C exhibit greater variability, likely due to lower class prevalence.
